# Trends in Levels of Cholesterol in Japanese Children from 1993 through 2001

**DOI:** 10.2188/jea.14.78

**Published:** 2005-03-18

**Authors:** Katsuyasu Kouda, Harunobu Nakamura, Rikio Tokunaga, Hiroichi Takeuchi

**Affiliations:** 1 Department of Hygiene, Kansai Medical University.; 2 Department of Public Health, Hamamatsu University School of Medicine.

**Keywords:** body mass index, hyperlipidemia, Japan, schools, cholesterol

## Abstract

BACKGROUND: Cardiovascular risk factors in children raise the possibility of cardiovascular disease later in life. We attempted to determine the current trends in cardiovascular risk factors among Japanese children.

METHODS: We examined fifth-graders at all the elementary schools in Iwata city in Japan every year from 1993 through 2001. We examined 4,673 boys and 4,484 girls, aged 10-11 years. Height, body weight, body mass index, and serum total cholesterol concentrations were measured. Regression analysis was used to evaluate the trends. The independent variable was the calendar year of the examination. The dependent variables were the anthropometric values and the serum total cholesterol concentration in each individual. The relationships between the year and the prevalence of hyperlipidemia and the prevalence of obesity were also examined.

RESULTS: Both the heights of the girls and the body weights of the both sexes were positively associated with the year. The body mass index in both girls and boys also showed positive relationships with the calendar year. In addition, there was an increase in the prevalence of obesity. Concerning the levels of cholesterol, positive regression coefficients were shown between the total cholesterol concentration and the year in both sexes. Furthermore, there was an increase in prevalence of hyperlipidemia.

CONCLUSION: These results indicate that during the past decade among Japanese children involved in the study, both the body mass index and the serum total cholesterol concentrations increased.

The incidence of coronary heart disease is linked to the prevalence of atherosclerosis in the general population. It has been reported that there is a tracking of serum lipid and lipoprotein concentrations from childhood to adulthood, and it is widely acknowledged that hyperlipidemia originates during youth.^1^^-^^4^ In autopsy studies, fatty arterial lesions have been found to develop in the early years of life.^5^^,^^6^ Furthermore, fatty streaks and clinically significant coronary lesions increase rapidly in both their prevalence and extent during adolescence and early adulthood.^7^

The incidence of coronary heart disease is also related to the prevalence of obesity. An association between the presence of obesity in childhood and obesity in adulthood has been reported in longitudinal studies.^8^^-^^10^ Furthermore, childhood obesity is related to adult levels of lipids, lipoproteins, blood pressure, and insulin and to morbidity from coronary heart disease.^11^ Therefore, recent trends in risk factors for cardiovascular disease in children are important predictors of subsequent trends in adults.

Annual changes in anthropometric values such as height and weight in Japanese children can be referred to in the nationwide report of school health statistics conducted by the Ministry of Education, Culture, Sports, Science, and Technology of the Japanese government.^12^ In addition, an increase has been reported in the prevalence of obesity in Japanese children.^13^^,^^14^ Increases in serum cholesterol concentrations have also been reported during the past 3 decades.^14^^-^^16^ However, these reports have not led to a clear determination of secular trends in serum cholesterol concentrations because of problems such as an absence of continuity in studies and the nonconformity of quality control and the precision of assessment in blood tests.

In the present study, we followed the whole city of Iwata in Japan from 1993 through 2001, and measured serum cholesterol levels in all school children aged 10-11 years, with consistency in the measurement protocol, and examined the regression analysis from the individual subject data to clarify the current trends of serum lipid concentrations among school-aged children. We also measured and examined the anthropometric values.

## METHODS

### Population

The city of Iwata is located in Shizuoka prefecture, Japan, 230 km from Tokyo. It covers an area of 64 km^2^ and in 2000 had a population of 86.7 thousand. Approximately 5 % of the city’s residents work in primary industries (agriculture, forestry, and fishing), 45 % work in secondary industries (mining, construction, and manufacturing), and 50 % work in tertiary industries (transportation, public utilities, wholesale or retail trade, finance, insurance, real estate, and services), while the average workforce proportions in Japanese communities are 7 % primary, 39 % secondary, and 54 % tertiary industries.

All fifth-graders (10-11 years of age) in all of the 11 elementary schools in Iwata were targeted for this study. We examined 99.9 % of the initially eligible children. A total of 9,157 children (4,673 boys and 4,484 girls) were examined. The sample sizes for each calendar year are listed in Table 1.

**Table 1.** Anthropometric variables in 10- to 11-year-old children, Iwata city, Japan.

	Survey year									Test for trends
	
	1993	1994	1995	1996	1997	1998	1999	2000	2001	Regression coefficient (95% CI)*
	Boys									
n	513	568	527	552	506	527	468	490	522	
Height (cm)										0.06 (-0.01, 0.13)
Mean	138.4	138.1	138.2	138.0	137.9	138.5	138.1	138.5	138.8	
Standard deviation	6.0	6.0	6.2	5.6	5.8	6.3	6.5	6.7	6.8	
Body weight (kg)										0.17 ( 0.09, 0.25)
Mean	33.7	33.2	33.3	33.5	33.4	34.0	34.0	34.4	34.9	
Standard deviation	7.0	6.5	6.4	6.1	6.7	6.8	7.6	8.4	7.9	

	Girls									
n	485	567	568	480	537	464	463	451	469	
Height (cm)										0.21 ( 0.13, 0.28)
Mean	138.8	138.7	139.0	139.1	139.8	140.0	139.8	140.2	140.2	
Standard deviation	6.4	6.4	6.6	6.7	6.5	6.5	6.5	7.0	7.1	
Body weight (kg)										0.17 ( 0.09, 0.25)
Mean	33.3	33.4	33.5	33.5	33.9	34.2	34.4	34.4	34.4	
Standard deviation	6.3	6.5	6.4	6.3	6.7	6.8	6.9	7.1	7.0	

CI: confidence interval

*: Simple regression analysis was used to evaluate trends. The independent variable was the year of the examination, and the dependent variables were the anthropometric values in each individual.

### Examinations

All examinations were conducted by the Iwata Board of Education during 3 months from April through June every year. Both the methodology and the instruments used remained unchanged throughout the study period, from 1993 through 2001. Measurements of height and body weight were made by *Yogo* teachers, who have a Japanese national educational license for health education and health care at the elementary school level. At each examination, height was measured to the closest 0.1 cm and weight to the closest 0.1 kg. Body mass index (BMI) was calculated by dividing weight (kg) by height (m) squared. The cutoff point of overweight and obesity was defined at 20.2 kg/m^2^ and 24.6 kg/m^2^ of BMI in boys, and 20.3 kg/m^2^ and 24.8 kg/m^2^ in girls, respectively.^17^ Blood samples were obtained by nurses and medical technologists. The serum total cholesterol (TC) was determined enzymatically (Pureauto CHO-N, Daiichi Pure Chemical Co., Ltd., Tokyo, Japan) using a Hitachi 7350 automatic chemistry analyzer. Accuracy and the precision of determination of serum TC were controlled by external quality assessment of the Japan Medical Association.

The present study was conducted with permission by the Iwata Board of Education. All the subjects received care in compliance with the ethical guidelines of the Declaration of Helsinki, and were protected from invasion of privacy.

### Statistical analysis

Statistical analysis was performed using SPSS^®^ Base 11.5J for Windows (SPSS Inc., Chicago, IL, USA). Simple regression analysis was used to evaluate the trends of each variable. The independent variable was the calendar year of the examination. The dependent variables were height, body weight, BMI, and TC in each individual. For the relationships between the year and the prevalence of hyperlipidemia or the prevalence of obesity, Mann-Whitney’s U test was used. A p value less than 0.05 was considered statistically significant.

## RESULTS

Both the heights of the girls and the body weights of the both sexes were significantly associated with the calendar year. The regression coefficient indicated that there was a height increase of 0.21 cm/year in girls, and a body-weight increase of 0.17 kg/year in both sexes (Table 1).

BMI in both girls and boys showed a significant relationship with the year. The regression coefficient indicated that there is a BMI increase of 0.07 kg/m^2^/year in boys and 0.03 kg/m^2^/year in girls. Furthermore, there were significant increases among prevalence of obesity (BMI≥20.2 kg/m^2^ in boys, ≥20.3 kg/m^2^ in girls) during the survey year in both sexes (Table 2).

**Table 2.** Body mass index (BMI) in 10- to 11-year-old children, Iwata city, Japan.

	Survey year									Test for trends
	
	1993	1994	1995	1996	1997	1998	1999	2000	2001	Regression coefficient (95% CI)*
	Boys									
Mean BMI (kg/m^2^)	17.5	17.3	17.4	17.5	17.5	17.6	17.7	17.8	18.0	0.07 (0.04, 0.10)
Standard deviation	2.6	2.5	2.4	2.5	2.5	2.6	2.9	3.0	2.9	
Number (%) of obesity										
Total	513	568	527	552	506	527	468	490	522	
BMI ≥ 20.2 kg/m^2^	69(13.5)	68(12.0)	61(11.6)	77(13.9)	59(11.7)	59(11.2)	67(14.3)	77(15.7)	77(14.8)	
BMI ≥ 24.6 kg/m^2^	10(1.9)	12(2.1)	9(1.7)	8(1.4)	13(2.6)	11(2.1)	15(3.2)	17(3.5)	21(4.0)	

	Girls									
Mean BMI (kg/m^2^)	17.2	17.3	17.2	17.2	17.2	17.3	17.5	17.4	17.4	0.03 (0.01, 0.06)
Standard deviation	2.3	2.4	2.3	2.2	2.4	2.5	2.5	2.5	2.6	
Number (%) of obesity										
Total	485	567	568	480	537	464	463	451	469	
BMI ≥ 20.3 kg/m^2^	48(9.9)	59(10.4)	49(8.6)	39(8.1)	52(9.7)	51(11.0)	61(13.2)	54(12.0)	57(12.2)	
BMI ≥ 24.8 kg/m^2^	4(0.8)	7(1.2)	6(1.1)	4(0.8)	8(1.5)	7(1.5)	7(1.5)	5(1.1)	10(2.1)	

CI: confidence interval

*: For evaluation of trends, simple regression analysis was used. The independent variable was the year of the examination, and the dependent variable was BMI in each individual.

There were significant increases among prevalence of obesity (BMI ≥ 20.2 kg/m^2^ in boys, ≥ 20.3 kg/m^2^ in girls) during the survey year in both sexes by Mann-Whitney’s U test.

Concerning TC concentrations, there were significant relationships with the year, with regression coefficients indicating increases of 0.02 mmol/L/year for TC values in both sexes. Furthermore, there were significant increases among prevalences of hyperlipidemia (TC≥5.17 mmol/L) during the survey year in both sexes (Table 3).

**Table 3.** Serum total cholesterol (TC) in 10- to 11-year-old children, Iwata city, Japan.

	Survey year									Test for trends
	
	1993	1994	1995	1996	1997	1998	1999	2000	2001	Regression coefficient (95% CI)*
	Boys									
Mean TC (mmol/L)	4.34	4.51	4.36	4.45	4.49	4.39	4.48	4.59	4.50	0.02 (0.01, 0.02)
Standard deviation	0.66	0.64	0.64	0.71	0.67	0.64	0.66	0.72	0.69	
Number (%) of hyperlipidemia										
Total	513	568	527	552	506	527	468	490	522	
TC ≥ 5.17 mmol/L	56(10.9)	88(15.5)	54(10.2)	84(15.2)	78(15.4)	63(12.0)	69(14.7)	94(19.2)	84(16.1)	
TC ≥ 5.69 mmol/L	14(2.7)	28(4.9)	18(3.4)	24(4.3)	27(5.3)	17(3.2)	19(4.1)	34(6.9)	25(4.8)	

	Girls									
Mean TC (mmol/L)	4.30	4.59	4.34	4.47	4.47	4.41	4.51	4.60	4.52	0.02 (0.01, 0.03)
Standard deviation	0.67	0.69	0.74	0.69	0.67	0.73	0.69	0.66	0.68	
Number (%) of hyperlipidemia										
Total	485	567	568	480	537	464	463	451	469	
TC ≥ 5.17 mmol/L	44(9.1)	114(20.1)	65(11.4)	64(13.3)	65(12.1)	67(14.4)	78(16.8)	96(21.3)	88(18.8)	
TC ≥ 5.69 mmol/L	13(2.7)	35(6.2)	15(2.6)	20(4.2)	17(3.2)	19(4.1)	20(4.3)	28(6.2)	26(5.5)	

CI: confidence interval

Total cholesterol; 5.17 mmol/L = 200 mg/dL, 5.69 mmol/L = 220 mg/dL

TC level (mg/dL) × 0.02586 = TC level (mmol/L)

*: For evaluation of trends, simple regression analysis was used. The independent variable was the year of the examination, and the dependent variable was levels of cholesterol in each individual.

There were significant increases among prevalence of hyperlipidemia (TC≥5.17 mmol/L) during the survey year in both sexes by Mann-Whitney’s U test.

## DISCUSSION

In the present study, trends in hyperlipidemia were investigated in all fifth-graders throughout the city of Iwata under a consistent protocol, with an increase in serum TC concentrations being found in both girls and boys. In the United States and European countries, there are established guidelines for reducing the risk of coronary heart disease by initiating intervention during childhood.^18^ It has been reported that fat intake in American children has decreased since the mid-1970s,^19^ and that serum cholesterol concentrations in American children have remained relatively stable over the past 2 or 3 decades.^19^^,^^20^ Among adults in the United States, mortality from coronary heart disease has declined since the mid-1960s.^21^^-^^23^ In Japan, on the other hand, there are no guidelines for interventions to reduce the risk of coronary heart disease in childhood. Previous studies of Japanese children have found that serum cholesterol concentrations have increased during the past 3 decades.^14^^-^^16^ As a result, Japanese children have higher serum cholesterol concentrations than American children of the same age. The mean serum cholesterol concentration in 9- to 10-year-old Japanese children was 4.01 mmol/L in 1960, 4.27 mmol/L in 1980, and 4.42 mmol/L in 1990, and the mean serum cholesterol concentration in 1990 exceeded the 75th percentile value of 4.40 mmol/L for American children.^16^ In addition, coronary heart disease has increased over the past few decades, and is now the second leading cause of death in Japan.^24^ Our results regarding current trends in TC concentrations among Japanese children are consistent with these previous reports.

In a nationwide report of school health statistics conducted by the Ministry of Education, Culture, Sports, Science, and Technology of the Japanese government,^12^ the mean height in boys was reported to be 138.8 cm in 1993 and 138.9 cm in 2001, and that in girls was reported to be 139.9 cm in 1993 and 140.3 cm in 2001. The mean body weight in boys was 34.3 kg in 1993 and 35.0 kg in 2001, and that in girls was 34.4 kg in 1993 and 34.7 kg in 2001. These data indicated a meaningful change in body weight in both sexes. The results of our regression analyses for the present population are consistent with the nationwide survey.

Although the change in the BMI can be analyzed using the mean value of anthropometrics described in the school of health statistics, we conducted the regression analyses by using individual subject data from the present study. The independent variable was the calendar year of the examination, and the dependent variable was the BMI. We found that the BMI showed a positive relationship with the examination year in both girls and boys. These results indicate that BMI has been increasing for the last decade. It has previously been reported that the prevalence of obesity in Japan in 1996 was more than twice that in 1970.^13^^,^^14^ These studies have hypothesized that the recent increases in the incidence of childhood obesity and hyperlipidemia in Japan are due to increased intake of fat and an increasingly sedentary lifestyle, including decreases in physical exercise and activity.^14^^,^^25^ Our result for BMI is consistent with these previous reports.

In conclusion, during the past decade there has been a significant increase in BMI and serum TC concentrations in children in Iwata city in Japan. Further study regarding the prevalence of obesity and hyperlipidemia in Japanese children is needed.
